# Cognitive and motor task performance under single- and dual-task conditions: effects of consecutive versus concurrent practice

**DOI:** 10.1007/s00221-021-06130-8

**Published:** 2021-06-18

**Authors:** Rainer Beurskens, Dennis Brueckner, Hagen Voigt, Thomas Muehlbauer

**Affiliations:** 1 Department of Health and Social Affairs, FHM Bielefeld - University of Applied Sciences, Bielefeld, Germany; 2 Division of Movement and Training Sciences/Biomechanics of Sport, University of Duisburg-Essen, Essen, Germany

**Keywords:** Skill acquisition, Stabilometer, Postural control, Auditory stroop task, Human

## Abstract

The concurrent execution of two or more tasks simultaneously results in performance decrements in one or both conducted tasks. The practice of dual-task (DT) situations has been shown to decrease performance decrements. The purpose of this study was to investigate the effects of consecutive versus concurrent practice on cognitive and motor task performance under single-task (ST) and DT conditions. Forty-five young adults (21 females, 24 males) were randomly assigned to either a consecutive practice (INT consecutive) group, a concurrent practice (INT concurrent) group or a control (CON) group (i.e., no practice). Both INT groups performed 2 days of acquisition, i.e., practicing a cognitive and a motor task either consecutively or concurrently. The cognitive task required participants to perform an auditory stroop task and the number of correct responses was used as outcome measure. In the motor task, participants were asked to stand on a stabilometer and to keep the platform as close to horizontal as possible. The time in balance was calculated for further analysis. Pre- and post-practice testing included performance assessment under ST (i.e., cognitive task only, motor task only) and DT (i.e., cognitive and motor task simultaneously) test conditions. Pre-practice testing revealed no significant group differences under ST and DT test conditions neither for the cognitive nor the motor task measure. During acquisition, both INT groups improved their cognitive and motor task performance. The post-practice testing showed significantly better cognitive and motor task values under ST and DT test conditions for the two INT groups compared to the CON group. Further comparisons between the two INT groups revealed better motor but not cognitive task values in favor of the INT consecutive practice group (ST: *p* = 0.022; DT: *p* = 0.002). We conclude that consecutive and concurrent practice resulted in better cognitive (ST condition) and motor (ST and DT test conditions) task performance than no practice. In addition, consecutive practice resulted in superior motor task performance (ST and DT test conditions) compared to concurrent practice and is, therefore, recommended when executing DT practice schedules.

## Introduction

The concurrent execution of two or more tasks simultaneously (i.e., performing a cognitive task and a motor tasks; dual-task (DT) condition) results in performance decrements in one or both concurrently conducted tasks in young adults (Krampe et al. 2011; Nurwulan et al. 2015; Raffegeau et al. 2018) as well as in older adults (Kerr et al. 1985; Ebersbach et al. 1995; Lundin-Olsson et al. 1997). Practicing DT situations have previously been shown to decrease these performance decrements during DT conditions in young adults (Pellecchia 2005; Kiss et al. 2018; Beurskens et al. 2020) and also in older adults (Silsupadol et al. 2009a, b). For example, Kiss et al. (2018) examined the effect of single-task (ST) versus DT practice on dynamic balance control in healthy young adults. Results showed that ST practice resulted in improvements of task performance in the trained domain (i.e., motor or cognitive performance) while DT practice showed an effective modulation of both domains (i.e., DT performance). Similar results were found by Beurskens et al. (2020) showing that DT but not ST practice resulted in an improved modulation of the motor as well as the cognitive domain during DT performance in young adults, irrespective of task prioritization.

However, when taking a closer look at the previously reported results, the experimental procedures used in many of the aforementioned studies compared DT practice (i.e., performing cognitive and motor task concurrently) to ST practice conditions where only one task was trained (i.e., performing the cognitive or the motor task only). Neither Beurskens et al. (2020) nor Kiss et al. (2018) trained both tasks (motor and cognitive) during ST practice independently in one group. Using this approach represents a weakness when comparing the effects of ST versus DT practice on DT performance and it is difficult to classify specific effects of the DT practice regimes. For ST practice to be comparable to the DT practice regimen, ST practice would need to incorporate both tasks during practice as well (i.e., practice the motor as well as the cognitive tasks consecutively). Thus, knowledge on using the appropriate practice schedule to improve DT performance following consecutive (i.e., practicing task A followed by practicing task B) or concurrent practice (i.e., simultaneous practice of task A and B) is needed.

To the authors’ knowledge, Pellecchia (2005) performed one of the few studies examining the effect of consecutive compared to concurrent practice on cognitive (i.e., serial three subtractions) and motor task performance in healthy adults using a relatively easy to administer balance task (i.e., quiet standing on a compliant surface). Results showed better motor task but not cognitive task performance in the concurrent practice but not in the consecutive training group when tested under DT condition. Concurrent practice might be superior to consecutive practice by integrating both tasks into practice (i.e., “task integration approach”) (Neumann 1987; Ruthruff et al. 2006) and performing the two tasks simultaneously, thereby causing improvement in DT situations. Hence, the performance of two tasks during DT is not an execution of two independent tasks at the same time but the performance of a new task that integrates and coordinates two individual subtasks into one superior DT. As a consequence, practice in DT situations is needed to improve DT performance by explicitly training subtasks and their coordination. In contrast, the “single channel model” (i.e., limited cognitive capacities) (Pashler 1994; Pashler and Johnston 1998) as well as the “capacity sharing model” (i.e., cognitive interference when two tasks share the same processing resources) (Tombu and Jolicoeur 2003; Wickens 2008) argue that consecutive practice is more effective than concurrent practice. Performance improvements during DT conditions might be triggered by improved task automatization which can be achieved by executing two tasks separately (i.e., practicing the motor task followed by practicing the cognitive task or vice versa). Accordingly, practicing two tasks independently until automatization (i.e., reduced attentional resources necessary) is needed to improve performance in DT situations. However, the most beneficial schedule of training the motor and cognitive task (i.e., consecutive vs. concurrent) remains unclear.

The aims of the present study were (a) to examine the effect of consecutive versus concurrent practice on cognitive and motor task performance under ST and DT test conditions in healthy young adults and (b) to adapt findings from Pellecchia (2005) to a more complex dynamic balance task. We expected that, irrespective of the practice regime (i.e., either consecutive or concurrent), cognitive and motor task performance would improve under ST and DT test conditions compared to no practice (i.e., control condition). In addition, we assumed with reference to the relevant literature (Pellecchia 2005) that the concurrent practice schedule would be more suitable to improve cognitive and motor task performance during DT test condition compared to the consecutive practice regimen, also in a more complex motor task.

## Materials and methods

### Participants and groups

Forty-five healthy young adults were randomly assigned to one of two active intervention (INT) groups or a passive control (CON) group. The INT consecutive practice group (*n* = 15; 8 men, 7 women; mean age: 23.4 ± 2.1 years) practiced the cognitive task first followed by the motor task. The INT concurrent practice group (*n* = 15; 8 men, 7 women; mean age: 25.7 ± 3.2 years) practiced the cognitive and the motor task simultaneously. The CON group (*n* = 15; 8 men, 7 women; mean age: 22.8 ± 2.5 years) did not practice either of the tasks. All participants had no prior experience with the experimental tasks and were not aware of the specific purpose of this study. All subjects signed informed consent forms prior to the experiment. The Human Ethics Committee at the University of Duisburg-Essen, Faculty of Educational Sciences approved the study protocol.

### Tasks

#### Cognitive task

**Fig. 1:**
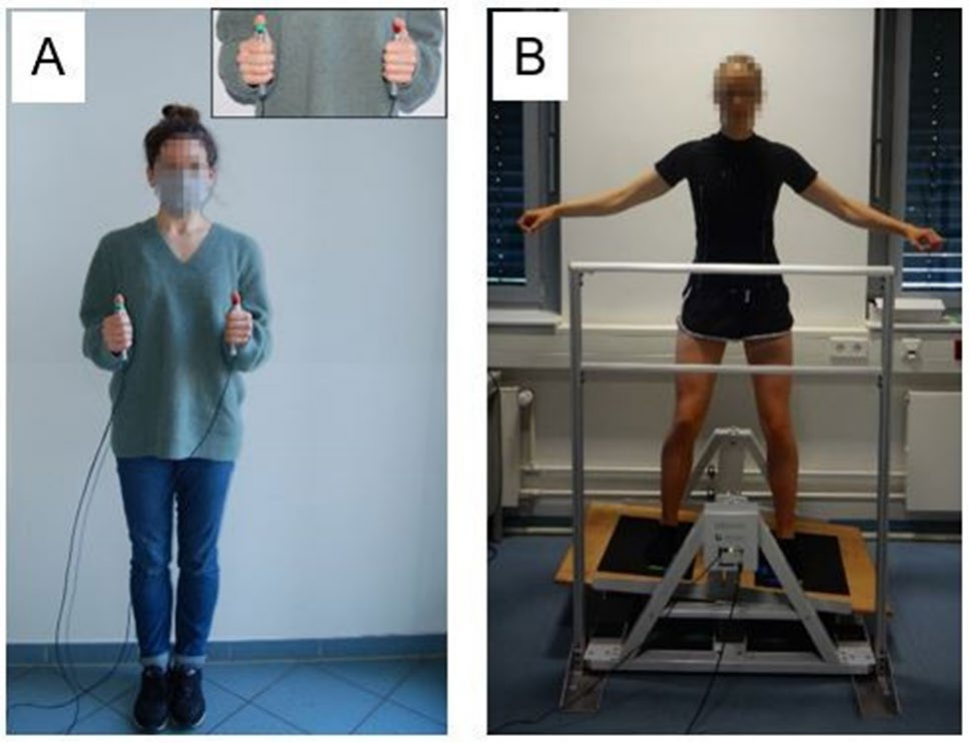
A participant performing the cognitive task (**A**) and the motor task (**B**) under single-task condition

The cognitive task used was an auditory stroop task (Morgan and Brandt 1989), in which the participants responded manually using two switches to two different pitches. More precisely, subjects heard the spoken words “high” and “low” that were presented in either a high or low pitch by a computer-generated voice. Participants were asked to ignore the actual word presented but to indicate the pitch of the word they heard by pressing the left (low pitch) or right (high pitch) switch during each 90-s trial (Fig. 1A). Low and high pitch were presented in compatible word/pitch (i.e., the spoken word “high” presented in a high pitch or the spoken word “low” presented in a low pitch) and in incompatible word/pitch combinations (i.e., the spoken word “high” presented in a low pitch or the spoken word “low” presented in a high pitch) in an equally distributed but randomized order. The number of correct responses was used as outcome measure. Thus, the higher the number was, the better the cognitive task performance.

#### Motor task

The motor task required participants to balance without shoes on a stability platform (Lafayette Instrument, Model 16,030, Lafayette, USA). The stability platform consisted of a 65 × 107-cm wooden platform and allowed a maximum deviation of 15 degrees from the horizontal to either side of the platform. Subjects were asked to remain in balance, i.e., to keep the platform in a horizontal position for as long as possible during each 90-s trial (Fig. 1B). A timer measured time in balance at a sampling rate of 25 Hz. Time in balance (s) was recorded when the platform was within ± 3 degrees of horizontal platform position and used as outcome measure (Taubert et al. 2016).

### Procedure

**Fig. 2:**
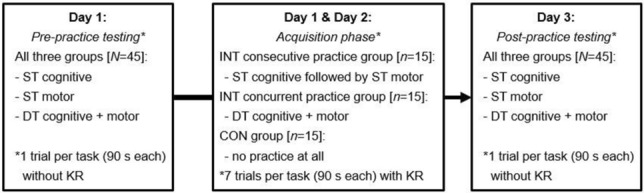
Schematic representation of the study design. *CON* control group, *DT *dual-task condition, *INT* intervention group, *KR* knowledge of result, *ST* single-task condition

The experimental design of the present study is displayed in Fig. 2. On day 1, all participants performed the following pre-practice testing sequence for one 90-s trial per task: (1) ST cognitive, (2) ST motor, and (3) DT cognitive + motor. Regarding the two ST conditions, instructions were as follows: “Please respond as accurately and as quickly as possible in the given trial” (ST cognitive) and “Please keep the platform as horizontal as possible and try to remain in a stable position in the given trial” (ST motor). Concerning the DT condition, instruction was as follows: “Please respond as accurately and as quickly as possible and keep the platform as horizontal as possible and try to remain in a stable position in the given trial” (DT cognitive + motor). Thereafter and on day 2, participants in INT consecutive practice group were instructed to perform seven 90-s practice trials for the cognitive task followed by seven 90-s trials for the motor task. Subjects in INT concurrent practice group performed the cognitive and the motor task simultaneously for seven 90-s trials on the 2 days. All trials were separated by a 90-s rest period to minimize possible fatigue effects. Both groups received knowledge of results (i.e., total number of correct response and/or time in balance) after the first, third, fifth, and seventh trials. Participants in CON group did neither practice the cognitive nor the motor task. On day 3, all participants conducted the post-practice testing using the same testing sequence as during pre-practice testing on day 1. The personnel involved in practice and in data analysis was blinded with respect to study hypotheses and the latter one was also blinded with respect to group affiliation.

### Statistical analyses

One-way analysis of variance (ANOVA) with additional post hoc comparisons (Bonferroni-corrected) was used to detect group differences during pre- and post-practice per testing condition (i.e., ST and DT testing). Further, a 2 (group: INT consecutive practice, INT concurrent practice) × 2 (day: day 1 and 2) × 7 (trial: trial 1 to 7) ANOVA with repeated measures on day and trial was used to assess group discrepancies during the acquisition phase. In addition, Cohen's *d* was calculated to determine whether a statistical difference was practically meaningful and was classified as small (0 ≤ *d* ≤ 0.49), medium (0.50 ≤ *d* ≤ 0.79), or large (*d* ≥ 0.80). All analyses were performed using the Statistical Package for Social Sciences (SPSS) version 27.0 and the significance level was set at *p* < 0.05.

## Results

### Pre-practice testing (day 1)

**Fig. 3:**
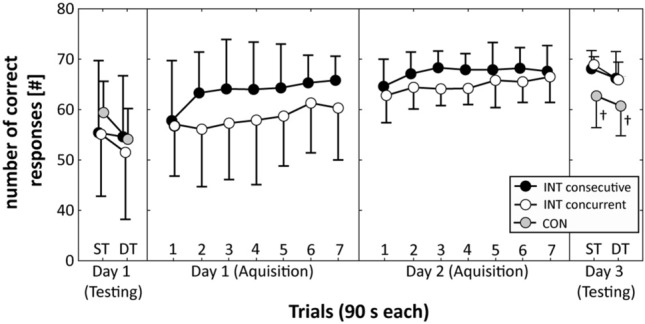
Total number of correct responses (i.e., cognitive task) of the two intervention (INT) groups and the control (CON) group during pre-practice testing (Day 1), acquisition phase (Day 1 and Day 2), and post-practice testing (Day 3). Values represent means and standard deviations. *ST* single-task test condition, *DT* dual-task test condition. †Indicates significantly lower values for the CON group compared to both INT groups

**Fig. 4:**
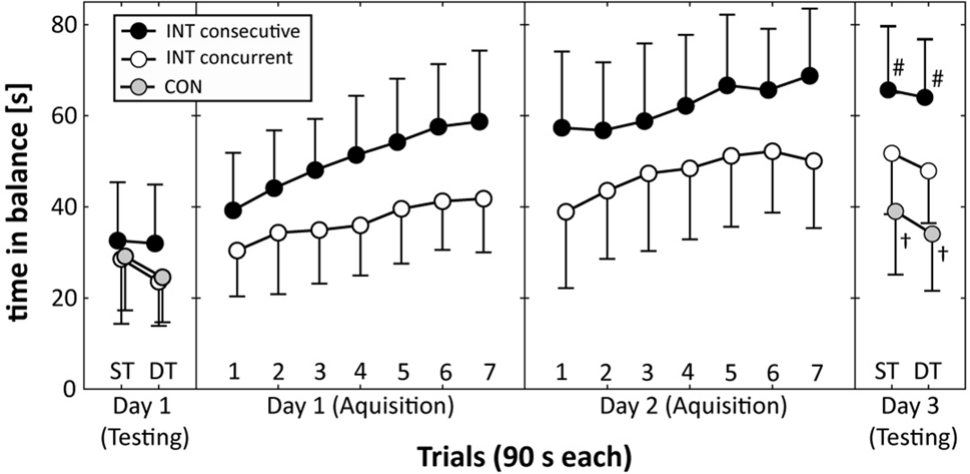
Time in balance (i.e., motor task) of the two intervention (INT) groups and the control (CON) group during pre-practice testing (Day 1), acquisition phase (Day 1 and Day 2), and post-practice testing (Day 3). Values represent means and standard deviations. *ST* single-task test condition, *DT* dual-task test condition. ^†^Indicates significantly lower values for the CON group compared to both INT groups. #Indicates significantly better values for the INT consecutive practice group compared to the INT concurrent practice group

Irrespective of testing condition (i.e., ST or DT), the one-way ANOVA showed no significant differences between the three groups, neither for the cognitive task [ST: *F*_(2,43)_ = 0.657, *p* = 0.523, *d* = 0.35; DT: *F*_(2,43)_ = 0.350, *p* = 0.707, *d* = 0.26] (Fig. 3) nor for the motor task [ST: *F*_(2,43)_ = 0.295, *p* = 0.746, *d* = 0.26; DT: *F*_(2, 43)_ = 2.117, *p* = 0.133, *d* = 0.67] (Fig. 4).

### Acquisition phase (day 1 and 2)

Figure 3 illustrates that both the INT consecutive practice group and the INT practice concurrent group enhanced their cognitive task performance (i.e., number of correct responses) over the two practice days. The Group × Day × Trial ANOVA yielded a statistically significant main effect of day [*F*_(1,28)_ = 13.247, *p* = 0.001, *d* = 1.35] and trial [*F*_(6,168)_ = 9.942, *p* < 0.001, *d* = 1.17] but not of group [*F*_(1,28)_ = 4.020, *p* = 0.054, *d* = 0.75]. The Group × Day × Trial interaction [*F*_(6,168)_ = 1.170, *p* = 0.324, *d* = 0.40] and the Day × Trial interaction [*F*_(6,168)_ = 1.247, *p* = 0.285, *d* = 0.41] did not reach the significance level.

As can be seen from Fig. 4, both practice groups improved their motor task performance (i.e., time in balance) across the two practice days. The Group × Day × Trial ANOVA revealed statistically significant main effects of day [*F*_(1,28)_ = 88.342, *p* < 0.001, *d* = 3.55], trial [*F*_(6,168)_ = 40.888, *p* < 0.001, *d* = 2.42], and group [*F*_(1,28)_ = 10.981, *p* = 0.003, *d* = 1.25]. The Group × Day × Trial interaction [*F*_(6,168)_ = 1.945, *p* = 0.076, *d* = 0.53] and the Day × Trial interaction [*F*_(6,168)_ = 0.848, *p* = 0.534, *d* = 0.35] did not reach the significance level.

### Post-practice testing (day 3)

For the cognitive task, one-way ANOVA showed significant differences between the three groups under ST [*F*_(2,43)_ = 9.416, *p* < 0.001, *d* = 1.32] and DT [*F*_(2,43)_ = 5.707, *p* = 0.006, *d* = 1.03] test conditions (Fig. 3). Post hoc comparisons for the ST test condition revealed larger numbers of correct responses for the INT consecutive practice group (*p* = 0.003) and the INT concurrent practice group (*p* = 0.001) compared to the CON group. No significant differences were found between the INT consecutive practice group and the INT concurrent practice group. Under DT test condition, post hoc comparisons showed significantly more numbers of correct responses for the INT consecutive practice group (*p* = 0.013) and the INT concurrent practice group (*p* = 0.021) than the CON group. Again, no significant differences were observed between the INT consecutive practice group and the INT concurrent practice group.

Regarding the motor task, one-way ANOVA revealed significant differences between the three groups under ST [*F*_(2,43)_ = 14.259, *p* < 0.001, *d* = 1.65] and DT [*F*_(2,43)_ = 22.192, *p* < 0.001, *d* = 2.08] conditions (Fig. 4). Under ST test conditions, post hoc comparisons yielded significantly longer times in balance for the INT consecutive practice group (*p* < 0.001) and the INT concurrent practice group (*p* = 0.047) compared to the CON group. In addition, the INT consecutive practice group outperformed (*p* = 0.022) the INT concurrent practice group. Under DT test condition, post hoc comparisons showed significantly longer times in balance for the INT consecutive practice group (*p* < 0.001) and the INT concurrent practice group (*p* = 0.012) than the CON group. Further, the INT consecutive practice group outperformed (*p* = 0.002) the INT concurrent practice group.

## Discussion

In the present study, we examined the effect of consecutive, concurrent or no practice on cognitive (i.e., auditory stroop task) and motor (i.e., dynamic balance task) task performance under ST and DT test conditions in healthy young adults. Our main findings can be summarized as follows: (1) both practice groups (i.e., INT consecutive practice and INT concurrent practice) improved their cognitive and motor task performance under ST and DT test conditions compared to the CON group and (2) the INT consecutive practice group showed better motor but not cognitive task performance than the INT concurrent practice group during ST and DT test conditions.

### Effects of practice on cognitive and motor task performance

The first hypothesis that irrespective of the practice regime (i.e., either consecutive or concurrent practice), cognitive and motor task performance will improve when tested under ST and DT conditions compared to no practice (i.e., control condition) was confirmed. These results correspond with previous findings from Worden and Vallis (2014), who investigated the impact of DT practice compared to ST practice or no practice on walking over either a static or dynamic obstacle (motor task) while responding to an auditory stroop task (cognitive task) in healthy young adults (mean age: 22.8 ± 2.1 years). The DT group practiced both tasks simultaneously, while the ST group practiced only the cognitive task, and the CON group received no practice. Besides others, the authors found that participants in the DT practice group but not in the CON group significantly improved their cognitive and motor task performance under DT test conditions.

### Effects of practice schedule on cognitive and motor task performance

Our second hypothesis that the concurrent practice schedule is better suited to improve cognitive and motor task performance during DT test condition was not confirmed. For the motor but not for the cognitive task, we detected performance differences depending on the practice schedule used. More precisely, our analyses showed significantly longer times in balance for the INT consecutive practice group compared to the INT concurrent practice group in ST as well as in DT test conditions*.* So far, only few studies investigated the effect of different practice conditions on cognitive and motor performance (Detweiler and Lundy 1995; Pellecchia 2005). For instance, Pellecchia (2005) examined the effect of consecutive compared to concurrent training or no training on cognitive (i.e., serial three subtractions) and balance (i.e., quiet standing on a compliant surface) performance in healthy adults aged 18–46 years. In contrast to our results, significantly better motor task performance in the concurrent but not in the consecutive training group when tested under DT condition was shown. Differences in the used methods may account for the discrepancies between our findings and those of Pellecchia (2005). More precisely, a static balance task (i.e., quiet standing) was used in the study of Pellecchia (2005) and a dynamic balance task (i.e., balancing on a moveable platform) was applied in the present study. There is evidence that static and dynamic components of balance are independent from each other (Muehlbauer et al. 2013) and thus different neuromuscular mechanisms seem to be responsible for the regulation of static and dynamic balance control. Further, only five 30-s trials per practice day were applied in the study of Pellecchia (2005), whereas seven 90-s trials per practice day were used in our study. This difference in the amount of practice might have caused a lower degree of task automatization in Pellecchia’s study (2005) compared to the present study, mitigating the requirements needed to apply the theories of limited resources (Pashler 1994; Pashler and Johnston 1998) or capacity sharing of attention (Tombu and Jolicoeur 2003; Wickens 2008). Similarly, Detweiler and Lundy (1995) also used a different approach of consecutive training compared to our study. The authors used a blocked design and trained the tasks alternating (i.e., task A followed by task B followed by task A, etc.) whereas in our study, tasks were presented in blocks of 7 (i.e., 7 trials of the cognitive task followed by 7 trials of the motor task). Further, the study by Detweiler and Lundy (1995) used two different visual tasks. That is, a task emphasizing verbal resources (word-category search task) and a task emphasizing spatial resources (spatial-pattern search task). The present study used a motor task and an auditory stroop task (Morgan and Brandt 1989). Hence, the discrepancy between our results and results from Pellecchia (2005) as well as from Detweiler and Lundy (1995) might indicate a dependency of task performance on the task used. Thus, authors of future studies are advised to consider the use of diverse motor/cognitive tasks in otherwise similar methodological approaches to address the question whether adaptation following DT practice is task-specific or task-unspecific.

In contrast to earlier studies on DT practice (Detweiler and Lundy 1995; Pellecchia 2005), our results indicate a higher efficacy of consecutive compared to concurrent practice conditions to improve DT task performance. The “single channel model” (Pashler 1994; Pashler and Johnston 1998) as well as the “capacity sharing model” (Tombu and Jolicoeur 2003; Wickens 2008) are well suited to explain our findings. Both models argue that limited cognitive resources are available during the execution of one or more tasks and these limited capacities have to be shared when two tasks involve the same processing resources and cognitive interference arises. During consecutive practice, two tasks are trained separately and automatization of both tasks is achieved, thereby also reducing interference between both tasks during DT condition. Thus, automatization of one or both tasks is needed to free up attentional resources during the execution of the given task. These additionally available resources can then be used during DT conditions to allocate more attentional resources to the simultaneous execution of both tasks. Consequently, a higher degree of automatization in a given task leads to better performances of parallel executed tasks and less interference during DT conditions. According to this interpretation, consecutive practice of tasks that lead to a higher degree of automatization (i.e., reduced attentional resources needed) is necessary to improve DT performance. This line of argumentation is also supported by findings from neurophysiological studies (Takeuchi et al. 2014; Garner and Dux 2015) showing functional as well as structural changes in the human brain following DT practice. For example, Takeuchi et al. (2014) showed increased gray matter volume in prefrontal cortical regions, the left posterior parietal cortex, and the left temporal and lateral occipital areas of the human brain following 4 weeks of multi-task training. Further, Garner and Dux (2015) were able to highlight specific regions in frontoparietal and subcortical areas of the brain, indicating increased activity in response to multi-task training. Based on the findings from Garner and Dux (2015) and our findings, DT practice in general and particularly consecutive DT practice seems suitable to trigger DT-specific neural adaptations enabling the brain to effectively adapt to DT demands.

## Conclusions

The present study examined the effect of consecutive, concurrent or no practice on cognitive and motor task performance under ST and DT test conditions in healthy young adults. Our findings suggest that irrespective of the practice regime, both cognitive and motor task performance improved when compared to no practice. Further, the type of practice schedule (consecutive or concurrent practice) differently affects cognitive and motor task performance with consecutive practice being more effective to improve motor but not cognitive task performance under ST and DT test conditions compared to concurrent practice.

## Data Availability

All data generated or analyzed during this study are included in this published article [and its supplementary information files].

## References

[CR1] Beurskens R, Brueckner D, Muehlbauer T (2020). Effects of motor versus cognitive task prioritization during dual-task practice on dual-task performance in young adults. Front Psychol.

[CR2] Detweiler MC, Lundy DH (1995). Effects of single and dual task practice on acquiring dual task skill. Hum Factors.

[CR3] Ebersbach G, Dimitrijevic MR, Poewe W (1995). Influence of concurrent tasks on gait: a dual-task approach. Percept Mot Skills.

[CR4] Garner KG, Dux PE (2015). Training conquers multitasking costs by dividing task representations in the frontoparietal-subcortical system. Proc Natl Acad Sci USA.

[CR5] Kerr B, Condon SM, McDonald LA (1985). Cognitive spatial processing and the regulation of posture. J Exp Psychol Hum Percept Perform.

[CR6] Kiss R, Brueckner D, Muehlbauer T (2018). Effects of single compared to dual task practice on learning a dynamic balance task in young adults. Front Psychol.

[CR7] Krampe RT, Schaefer S, Lindenberger U, Baltes PB (2011). Lifespan changes in multi-tasking: concurrent walking and memory search in children, young, and older adults. Gait Posture.

[CR8] Lundin-Olsson L, Nyberg L, Gustafson Y (1997). “Stops walking when talking” as a predictor of falls in elderly people. Lancet.

[CR9] Morgan AL, Brandt JF (1989). An auditory stroop effect for pitch, loudness, and time. Brain Lang.

[CR10] Muehlbauer T, Gollhofer A, Granacher U (2013). Association of balance, strength, and power measures in young adults. J Strength Cond Res.

[CR11] Neumann O, Heuer H, Sanders AF (1987). Beyond capacity: a functional view of attention. Perspectives on perception and action.

[CR12] Nurwulan NR, Jiang BC, Iridiastadi H (2015). Posture and texting: effect on balance in young adults. PLoS ONE.

[CR13] Pashler H (1994). Dual-task interference in simple tasks: data and theory. Psychol Bull.

[CR14] Pashler H, Johnston JC, Pashler H (1998). Attentional limitations in dual-task performance. Attention.

[CR15] Pellecchia GL (2005). Dual-task training reduces impact of cognitive task on postural sway. J Mot Behav.

[CR16] Raffegeau TE, Haddad JM, Huber JE, Rietdyk S (2018). Walking while talking: young adults flexibly allocate resources between speech and gait. Gait Posture.

[CR17] Ruthruff E, Van Selst M, Johnston JC, Remington R (2006). How does practice reduce dual-task interference: integration, automatization, or just stage-shortening?. Psychol Res.

[CR18] Silsupadol P, Lugade V, Shumway-Cook A, van Donkelaar P, Chou LS, Mayr U, Woollacott MH (2009). Training-related changes in dual-task walking performance of elderly persons with balance impairment: a double-blind, randomized controlled trial. Gait Posture.

[CR19] Silsupadol P, Shumway-Cook A, Lugade V, van Donkelaar P, Chou LS, Mayr U, Woollacott MH (2009). Effects of single-task versus dual-task training on balance performance in older adults: a double-blind, randomized controlled trial. Arch Phys Med Rehabil.

[CR20] Takeuchi H, Taki Y, Nouchi R (2014). Effects of multitasking-training on gray matter structure and resting state neural mechanisms. Hum Brain Mapp.

[CR21] Taubert M, Mehnert J, Pleger B, Villringer A (2016). Rapid and specific gray matter changes in M1 induced by balance training. Neuroimage.

[CR22] Tombu M, Jolicoeur P (2003). A central capacity sharing model of dual-task performance. J Exp Psychol Hum Percept Perform.

[CR23] Wickens CD (2008). Multiple resources and mental workload. Hum Factors.

[CR24] Worden TA, Vallis LA (2014). Concurrent performance of a cognitive and dynamic obstacle avoidance task: influence of dual-task training. J Mot Behav.

